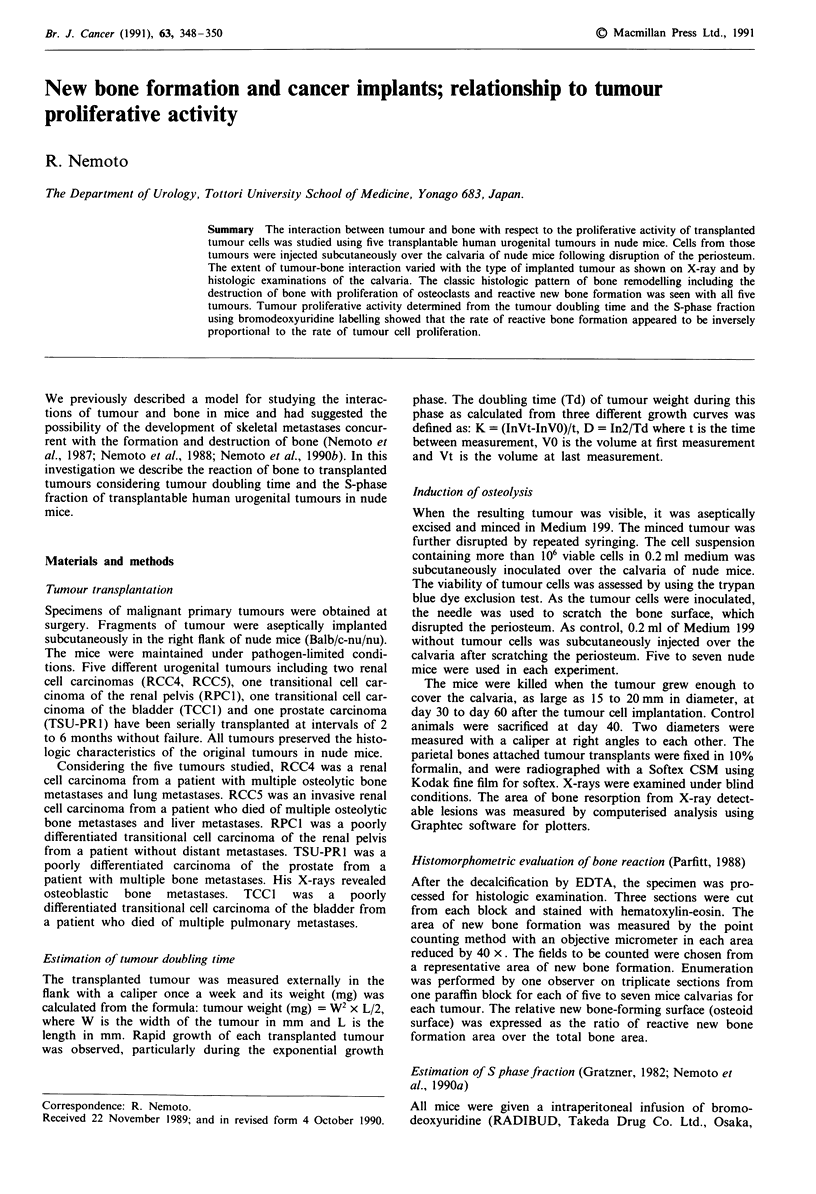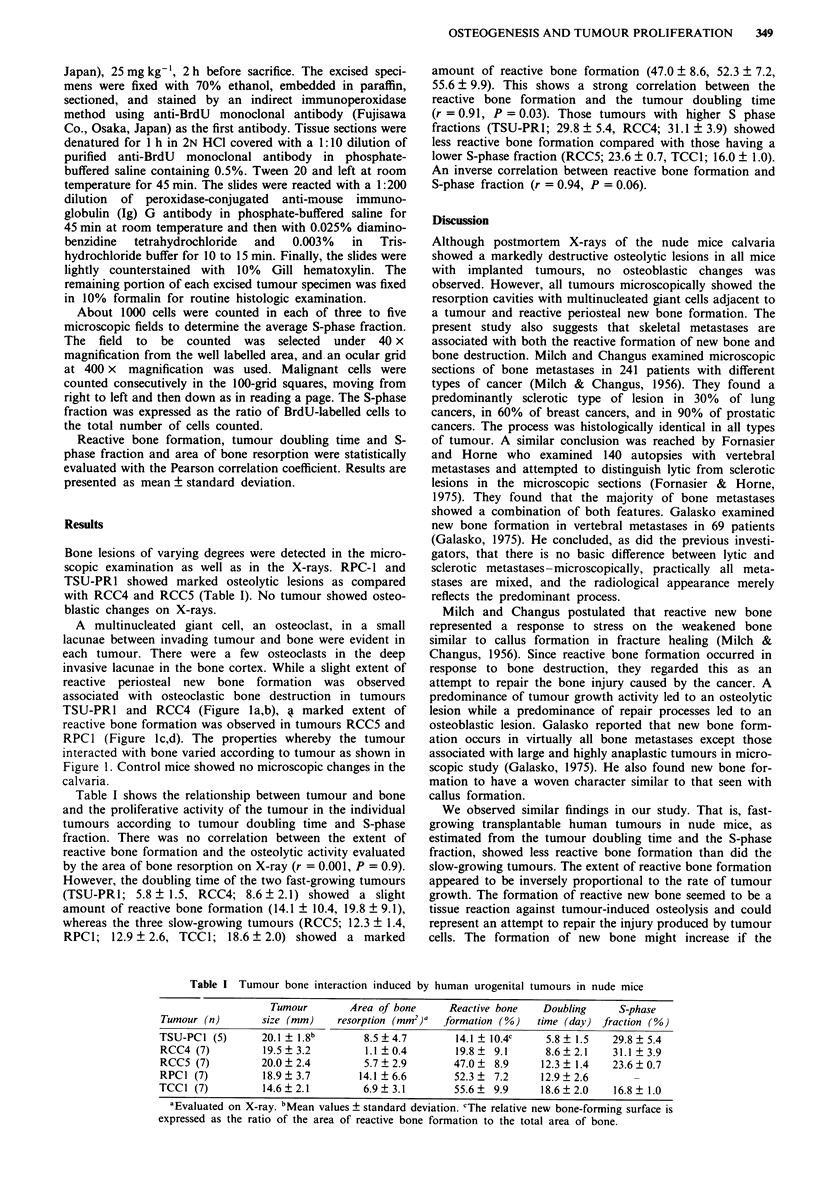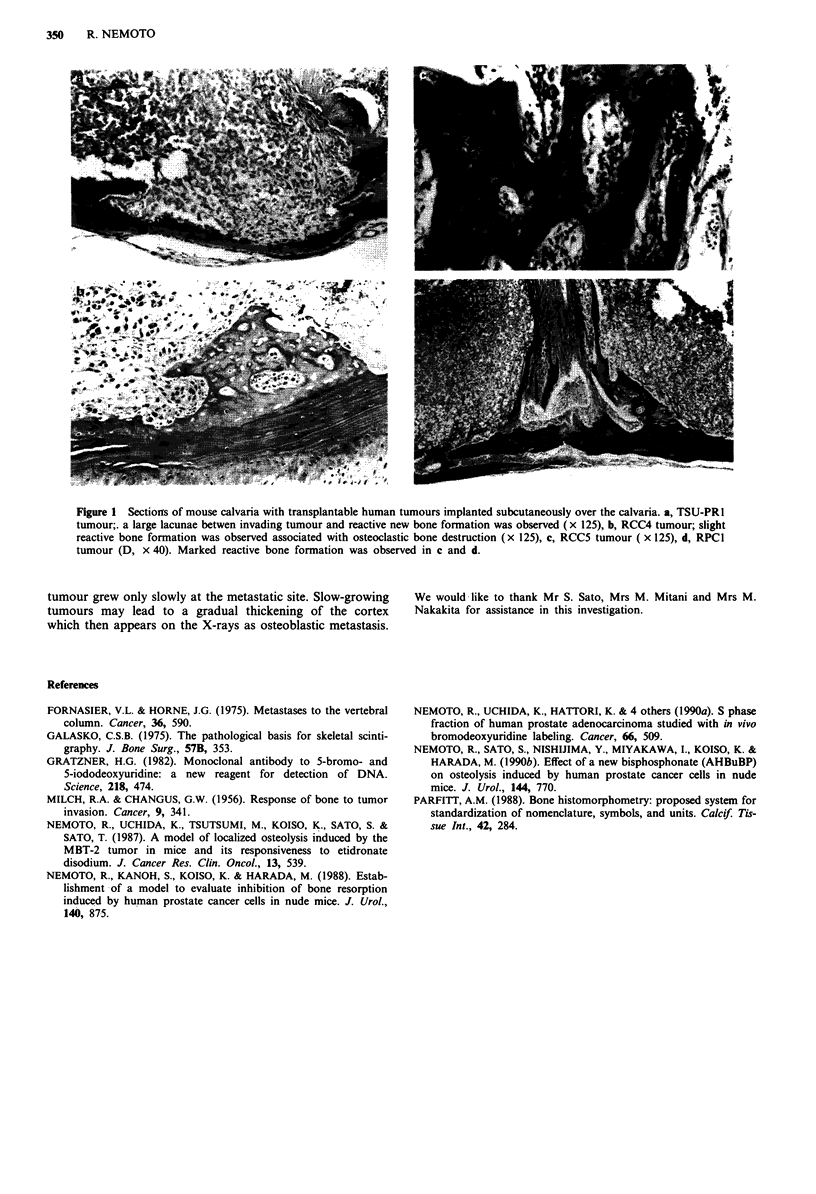# New bone formation and cancer implants; relationship to tumour proliferative activity.

**DOI:** 10.1038/bjc.1991.83

**Published:** 1991-03

**Authors:** R. Nemoto

**Affiliations:** Department of Urology, Tottori University School of Medicine, Yonago, Japan.

## Abstract

**Images:**


Br. J. Cancer (1991), 63, 348–350 © Macmillan Press Ltd., 1991

### New bone formation and cancer implants; relationship to tumour proliferative activity

R. Nemoto

*The Department of Urology, Tottori University School of Medicine, Yonago 683, Japan.*

**Summary** The interaction between tumour and bone with respect to the proliferative activity of transplanted tumour cells was studied using five transplantable human urogenital tumours in nude mice. Cells from those tumours were injected subcutaneously over the calvaria of nude mice following disruption of the periosteum. The extent of tumour-bone interaction varied with the type of implanted tumour as shown on X-ray and by histologic examinations of the calvaria. The classic histologic pattern of bone remodelling including the destruction of bone with proliferation of osteoclasts and reactive new bone formation was seen with all five tumours. Tumour proliferative activity determined from the tumour doubling time and the S-phase fraction using bromodeoxyuridine labelling showed that the rate of reactive bone formation appeared to be inversely proportional to the rate of tumour cell proliferation.

We previously described a model for studying the interactions of tumour and bone in mice and had suggested the possibility of the development of skeletal metastases concurrent with the formation and destruction of bone (Nemoto *et al.*, 1987; Nemoto *et al.*, 1988; Nemoto *et al.*, 1990*b*). In this investigation we describe the reaction of bone to transplanted tumours considering tumour doubling time and the S-phase fraction of transplantable human urogenital tumours in nude mice.

### Materials and methods

### *Tumour transplantation*

Specimens of malignant primary tumours were obtained at surgery. Fragments of tumour were aseptically implanted subcutaneously in the right flank of nude mice (Balb/c-nu/nu). The mice were maintained under pathogen-limited conditions. Five different urogenital tumours including two renal cell carcinomas (RCC4, RCC5), one transitional cell carcinoma of the renal pelvis (RPC1), one transitional cell carcinoma of the bladder (TCC1) and one prostate carcinoma (TSU-PR1) have been serially transplanted at intervals of 2 to 6 months without failure. All tumours preserved the histologic characteristics of the original tumours in nude mice.

Considering the five tumours studied, RCC4 was a renal cell carcinoma from a patient with multiple osteolytic bone metastases and lung metastases. RCC5 was an invasive renal cell carcinoma from a patient who died of multiple osteolytic bone metastases and liver metastases. RPC1 was a poorly differentiated transitional cell carcinoma of the renal pelvis from a patient without distant metastases. TSU-PR1 was a poorly differentiated carcinoma of the prostate from a patient with multiple bone metastases. His X-rays revealed osteoblastic bone metastases. TCC1 was a poorly differentiated transitional cell carcinoma of the bladder from a patient who died of multiple pulmonary metastases.

### *Estimation of tumour doubling time*

The transplanted tumour was measured externally in the flank with a caliper once a week and its weight (mg) was calculated from the formula: tumour weight (mg) = $W^2 \times L/2$, where W is the width of the tumour in mm and L is the length in mm. Rapid growth of each transplanted tumour was observed, particularly during the exponential growth phase. The doubling time (Td) of tumour weight during this phase as calculated from three different growth curves was defined as: $K = (\text{InVt}-\text{InV0})/t$, $D = \text{In}2/\text{Td}$ where t is the time between measurement, V0 is the volume at first measurement and Vt is the volume at last measurement.

### *Induction of osteolysis*

When the resulting tumour was visible, it was aseptically excised and minced in Medium 199. The minced tumour was further disrupted by repeated syringing. The cell suspension containing more than $10^6$ viable cells in 0.2 ml medium was subcutaneously inoculated over the calvaria of nude mice. The viability of tumour cells was assessed by using the trypan blue dye exclusion test. As the tumour cells were inoculated, the needle was used to scratch the bone surface, which disrupted the periosteum. As control, 0.2 ml of Medium 199 without tumour cells was subcutaneously injected over the calvaria after scratching the periosteum. Five to seven nude mice were used in each experiment.

The mice were killed when the tumour grew enough to cover the calvaria, as large as 15 to 20 mm in diameter, at day 30 to day 60 after the tumour cell implantation. Control animals were sacrificed at day 40. Two diameters were measured with a caliper at right angles to each other. The parietal bones attached tumour transplants were fixed in 10% formalin, and were radiographed with a Softex CSM using Kodak fine film for softex. X-rays were examined under blind conditions. The area of bone resorption from X-ray detectable lesions was measured by computerised analysis using Graphtec software for plotters.

### *Histomorphometric evaluation of bone reaction* (Parfitt, 1988)

After the decalcification by EDTA, the specimen was processed for histologic examination. Three sections were cut from each block and stained with hematoxylin-eosin. The area of new bone formation was measured by the point counting method with an objective micrometer in each area reduced by 40 ×. The fields to be counted were chosen from a representative area of new bone formation. Enumeration was performed by one observer on triplicate sections from one paraffin block for each of five to seven mice calvarias for each tumour. The relative new bone-forming surface (osteoid surface) was expressed as the ratio of reactive new bone formation area over the total bone area.

### *Estimation of S phase fraction* (Gratzner, 1982; Nemoto *et al.*, 1990*a*)

All mice were given a intraperitoneal infusion of bromodeoxyuridine (RADIBUD, Takeda Drug Co. Ltd., Osaka,

Correspondence: R. Nemoto.
Received 22 November 1989; and in revised form 4 October 1990.

Japan), 25 mg kg$^{-1}$, 2 h before sacrifice. The excised specimens were fixed with 70% ethanol, embedded in paraffin, sectioned, and stained by an indirect immunoperoxidase method using anti-BrdU monoclonal antibody (Fujisawa Co., Osaka, Japan) as the first antibody. Tissue sections were denatured for 1 h in 2N HCl covered with a 1:10 dilution of purified anti-BrdU monoclonal antibody in phosphate-buffered saline containing 0.5%. Tween 20 and left at room temperature for 45 min. The slides were reacted with a 1:200 dilution of peroxidase-conjugated anti-mouse immunoglobulin (Ig) G antibody in phosphate-buffered saline for 45 min at room temperature and then with 0.025% diaminobenzidine tetrahydrochloride and 0.003% in Tris-hydrochloride buffer for 10 to 15 min. Finally, the slides were lightly counterstained with 10% Gill hematoxylin. The remaining portion of each excised tumour specimen was fixed in 10% formalin for routine histologic examination.

About 1000 cells were counted in each of three to five microscopic fields to determine the average S-phase fraction. The field to be counted was selected under 40 × magnification from the well labelled area, and an ocular grid at 400 × magnification was used. Malignant cells were counted consecutively in the 100-grid squares, moving from right to left and then down as in reading a page. The S-phase fraction was expressed as the ratio of BrdU-labelled cells to the total number of cells counted.

Reactive bone formation, tumour doubling time and S-phase fraction and area of bone resorption were statistically evaluated with the Pearson correlation coefficient. Results are presented as mean ± standard deviation.

### Results

Bone lesions of varying degrees were detected in the microscopic examination as well as in the X-rays. RPC-1 and TSU-PR1 showed marked osteolytic lesions as compared with RCC4 and RCC5 (Table I). No tumour showed osteoblastic changes on X-rays.

A multinucleated giant cell, an osteoclast, in a small lacunae between invading tumour and bone were evident in each tumour. There were a few osteoclasts in the deep invasive lacunae in the bone cortex. While a slight extent of reactive periosteal new bone formation was observed associated with osteoclastic bone destruction in tumours TSU-PR1 and RCC4 (Figure 1a,b), a marked extent of reactive bone formation was observed in tumours RCC5 and RPC1 (Figure 1c,d). The properties whereby the tumour interacted with bone varied according to tumour as shown in Figure 1. Control mice showed no microscopic changes in the calvaria.

Table I shows the relationship between tumour and bone and the proliferative activity of the tumour in the individual tumours according to tumour doubling time and S-phase fraction. There was no correlation between the extent of reactive bone formation and the osteolytic activity evaluated by the area of bone resorption on X-ray ($r$ = 0.001, $P$ = 0.9). However, the doubling time of the two fast-growing tumours (TSU-PR1; 5.8 ± 1.5, RCC4; 8.6 ± 2.1) showed a slight amount of reactive bone formation (14.1 ± 10.4, 19.8 ± 9.1), whereas the three slow-growing tumours (RCC5; 12.3 ± 1.4, RPC1; 12.9 ± 2.6, TCC1; 18.6 ± 2.0) showed a marked amount of reactive bone formation (47.0 ± 8.6, 52.3 ± 7.2, 55.6 ± 9.9). This shows a strong correlation between the reactive bone formation and the tumour doubling time ($r$ = 0.91, $P$ = 0.03). Those tumours with higher S phase fractions (TSU-PR1; 29.8 ± 5.4, RCC4; 31.1 ± 3.9) showed less reactive bone formation compared with those having a lower S-phase fraction (RCC5; 23.6 ± 0.7, TCC1; 16.0 ± 1.0). An inverse correlation between reactive bone formation and S-phase fraction ($r$ = 0.94, $P$ = 0.06).

### Discussion

Although postmortem X-rays of the nude mice calvaria showed a markedly destructive osteolytic lesions in all mice with implanted tumours, no osteoblastic changes was observed. However, all tumours microscopically showed the resorption cavities with multinucleated giant cells adjacent to a tumour and reactive periosteal new bone formation. The present study also suggests that skeletal metastases are associated with both the reactive formation of new bone and bone destruction. Milch and Changus examined microscopic sections of bone metastases in 241 patients with different types of cancer (Milch & Changus, 1956). They found a predominantly sclerotic type of lesion in 30% of lung cancers, in 60% of breast cancers, and in 90% of prostatic cancers. The process was histologically identical in all types of tumour. A similar conclusion was reached by Fornasier and Horne who examined 140 autopsies with vertebral metastases and attempted to distinguish lytic from sclerotic lesions in the microscopic sections (Fornasier & Horne, 1975). They found that the majority of bone metastases showed a combination of both features. Galasko examined new bone formation in vertebral metastases in 69 patients (Galasko, 1975). He concluded, as did the previous investigators, that there is no basic difference between lytic and sclerotic metastases–microscopically, practically all metastases are mixed, and the radiological appearance merely reflects the predominant process.

Milch and Changus postulated that reactive new bone represented a response to stress on the weakened bone similar to callus formation in fracture healing (Milch & Changus, 1956). Since reactive bone formation occurred in response to bone destruction, they regarded this as an attempt to repair the bone injury caused by the cancer. A predominance of tumour growth activity led to an osteolytic lesion while a predominance of repair processes led to an osteoblastic lesion. Galasko reported that new bone formation occurs in virtually all bone metastases except those associated with large and highly anaplastic tumours in microscopic study (Galasko, 1975). He also found new bone formation to have a woven character similar to that seen with callus formation.

We observed similar findings in our study. That is, fast-growing transplantable human tumours in nude mice, as estimated from the tumour doubling time and the S-phase fraction, showed less reactive bone formation than did the slow-growing tumours. The extent of reactive bone formation appeared to be inversely proportional to the rate of tumour growth. The formation of reactive new bone seemed to be a tissue reaction against tumour-induced osteolysis and could represent an attempt to repair the injury produced by tumour cells. The formation of new bone might increase if the

**Table I** Tumour bone interaction induced by human urogenital tumours in nude mice

| *Tumour (n)* | *Tumour size (mm)* | *Area of bone resorption (mm²)*[a] | *Reactive bone formation (%)* | *Doubling time (day)* | *S-phase fraction (%)* |
|---|---|---|---|---|---|
| TSU-PC1 (5) | 20.1 ± 1.8[b] | 8.5 ± 4.7 | 14.1 ± 10.4[c] | 5.8 ± 1.5 | 29.8 ± 5.4 |
| RCC4 (7) | 19.5 ± 3.2 | 1.1 ± 0.4 | 19.8 ± 9.1 | 8.6 ± 2.1 | 31.1 ± 3.9 |
| RCC5 (7) | 20.0 ± 2.4 | 5.7 ± 2.9 | 47.0 ± 8.9 | 12.3 ± 1.4 | 23.6 ± 0.7 |
| RPC1 (7) | 18.9 ± 3.7 | 14.1 ± 6.6 | 52.3 ± 7.2 | 12.9 ± 2.6 | – |
| TCC1 (7) | 14.6 ± 2.1 | 6.9 ± 3.1 | 55.6 ± 9.9 | 18.6 ± 2.0 | 16.8 ± 1.0 |

[a]Evaluated on X-ray. [b]Mean values ± standard deviation. [c]The relative new bone-forming surface is expressed as the ratio of the area of reactive bone formation to the total area of bone.

**Figure 1** Sections of mouse calvaria with transplantable human tumours implanted subcutaneously over the calvaria. **a**, TSU-PR1 tumour;. a large lacunae betwen invading tumour and reactive new bone formation was observed (× 125), **b**, RCC4 tumour; slight reactive bone formation was observed associated with osteoclastic bone destruction (× 125), **c**, RCC5 tumour (× 125), **d**, RPC1 tumour (D, × 40). Marked reactive bone formation was observed in **c** and **d**.

tumour grew only slowly at the metastatic site. Slow-growing tumours may lead to a gradual thickening of the cortex which then appears on the X-rays as osteoblastic metastasis.

We would like to thank Mr S. Sato, Mrs M. Mitani and Mrs M. Nakakita for assistance in this investigation.

### References

FORNASIER, V.L. & HORNE, J.G. (1975). Metastases to the vertebral column. *Cancer*, **36**, 590.

GALASKO, C.S.B. (1975). The pathological basis for skeletal scintigraphy. *J. Bone Surg.*, **57B**, 353.

GRATZNER, H.G. (1982). Monoclonal antibody to 5-bromo- and 5-iododeoxyuridine: a new reagent for detection of DNA. *Science*, **218**, 474.

MILCH, R.A. & CHANGUS, G.W. (1956). Response of bone to tumor invasion. *Cancer*, **9**, 341.

NEMOTO, R., UCHIDA, K., TSUTSUMI, M., KOISO, K., SATO, S. & SATO, T. (1987). A model of localized osteolysis induced by the MBT-2 tumor in mice and its responsiveness to etidronate disodium. *J. Cancer Res. Clin. Oncol.*, **13**, 539.

NEMOTO, R., KANOH, S., KOISO, K. & HARADA, M. (1988). Establishment of a model to evaluate inhibition of bone resorption induced by human prostate cancer cells in nude mice. *J. Urol.*, **140**, 875.

NEMOTO, R., UCHIDA, K., HATTORI, K. & 4 others (1990*a*). S phase fraction of human prostate adenocarcinoma studied with *in vivo* bromodeoxyuridine labeling. *Cancer*, **66**, 509.

NEMOTO, R., SATO, S., NISHIJIMA, Y., MIYAKAWA, I., KOISO, K. & HARADA, M. (1990*b*). Effect of a new bisphosphonate (AHBuBP) on osteolysis induced by human prostate cancer cells in nude mice. *J. Urol.*, **144**, 770.

PARFITT, A.M. (1988). Bone histomorphometry: proposed system for standardization of nomenclature, symbols, and units. *Calcif. Tissue Int.*, **42**, 284.